# Reconfigurable polarization processor based on coherent four-port micro-ring resonator

**DOI:** 10.1515/nanoph-2023-0031

**Published:** 2023-10-23

**Authors:** Dan Yi, Jiapeng Luan, Yi Wang, Hon Ki Tsang

**Affiliations:** Department of Electronic Engineering, The Chinese University of Hong Kong, Hong Kong, China

**Keywords:** micro-ring resonator, polarization descrambler, polarization switching, polarization analyzer

## Abstract

Polarization processors with versatile functionalities are needed in optical systems, which use or manipulate polarized light. In this paper, we propose and realize an integrated polarization processor based on a coherent 4-port micro-ring resonator. The arbitrary unknown polarization state is input to the polarization processor via a 2-dimensional grating coupler (2DGC), which serves as a polarization beam splitter. The coherent 4-port micro-ring resonator (MRR) operates as a unitary processor and is formed by one crossbar micro-ring resonator and two thermally tunable phase shifters, one of which tunes the micro-ring while the other tunes the coherent interference between the two inputs from the 2DGC. The 4-port system can be used to control the input polarization states that appear at the two output ports and, therefore, can be used to implement a multi-function polarization processor, including polarization descrambler, polarization switch, polarizers, and polarization analyzer (both division of space (DOS) and division of time (DOT)). In this paper, we experimentally demonstrate the use of coherent 4-port MRR for polarization mode switching and for polarization mode unscrambling. The polarization unscrambler was capable of separating two polarization-multiplexed 40 GHz data lanes from the input fiber with crosstalk levels below −21 dB and is suitable for use in the receiver for polarization-multiplexed direct-detection optical communications systems. The same photonic circuit may be used as a polarization analyzer, either as a DOS polarization analyzer or a DOT polarization analyzer. The DOS polarization analyzer measured the polarization with measured deviation of the orientation angle (2*ψ*) varying from −0.5° to 1.3°and deviation of ellipticity angle (2*χ*) varying from −0.98° to 7.27°. The DOT polarization analyzer measured the polarization with a deviation of the orientation angle (2*ψ*) that varied from −2.93° to 3.49° and deviation of ellipticity angle (2*χ*) that varied from −3.5° to 3.05°.

## 1 Introduction

Silicon photonics is now a mainstream platform for photonic-integrated circuits because of its advantages, stemming from its high index-contrast waveguides, which allow tight bends for compact photonic circuits, and the use of the mature CMOS fabrication technology and supply chain, offering the proven scalability to large-volume low-cost manufacturing and high-yield integration and packaging [1–3]. Polarization-multiplexed (pol-mux) optical fiber communications are already widely deployed in long-haul coherent optical communications, enabling a doubling of the information carrying capacity of long distance optical fiber networks [4–7] while relying on the high-speed digital signal processing (DSP) needed in coherent optical communication systems for recovering and demultiplexing the different data lanes transmitted in orthogonal polarizations. Polarization manipulation is also needed in quantum information processing [8–11], optical sensing, such as in target detection [12, 13], and geological events observation [14, 15] and wideband test equipment, which use nonpolarization maintaining optical fibers for testing polarization-dependent devices. Polarized light is typically scrambled by transmission in standard single mode optical fibers and the waveguide birefringence [1] of the silicon-on-insulator nanowire platform, which leads to polarization mode dispersion, polarization-dependent loss, and polarization-dependent wavelength characteristics [16]. To recover the original polarization basis states without the use of high power-consumption high-speed DSP, one possible approach involves the use of a polarization diversity receiver, which captures two arbitrary orthogonal polarization states (not necessarily the original polarization basis) and process the orthogonal polarizations optically using polarizers [17–19], polarization beam splitters [16, 20–23], polarization rotators [24–26], polarization switches [27–32], and variable phase shifters. Polarization processors that can tune both the phase difference and the ratio of the two orthogonal polarization components can be used to recover the original polarization basis states and offer a more power-efficient and lower-latency solution for direct detection communication systems, which do not require the use of high-speed DSP for recovering the complex amplitudes of the optical fields that is needed in coherent optical communications [4–7]. The ability to dynamically adapt to changes in the state of polarization is important and useful because the SOP can vary rapidly under conditions such as mechanical vibrations, strain in the optical cables, change of temperature, or other disturbances in the optical fiber [33–35]. The random selection of two arbitrary orthogonal polarization states at the receiver will in general introduce polarization crosstalk between the original data channels. Full recovery of the transmitted optical fields in a coherent receiver typically uses the polarization tracking capability in the DSP algorithms. Pol-mux communications has not yet been deployed in direct-detection optical fiber communications links short reach data center interconnects because of the high-power consumption of high-speed coherent DSP, and there is not yet a deployable solution to recover the original polarization data channels in direct detection pol-mux communication links. The ability to monitor the SOP of polarized light is also required for optical sensing systems [12–15], which utilize the SOP as an indicator of various external perturbations to the fiber. On-chip polarization analyzers used for SOP monitoring have been previously developed based on Stokes vector receivers [36, 37], photonic crystal-like meta-structure [38], surface plasmon polaritons [39], Mach–Zehnder interferometers (MZI) [27], and MRR [40]. In this paper, we propose and demonstrate reconfigurable polarization processors, which can be used to control and monitor of SOP after transmission in an optical fiber. One approach to recover the original polarization basis states is via the use of a coherent network of MZI processors [27]. Coherent MZI networks can perform matrix operations and have found applications in deep learning [41], quantum information processing [42], and spatial modes unscrambling [43]. Recently, we proposed the use of coherent MRR networks as an alternative to the MZI for unitary matrix operations, with the advantages of energy efficiency, smaller latency, and more compact size [44].

In this paper, we propose and demonstrate a reconfigurable silicon photonics–integrated polarization processor. The device comprises a coherent 4-port MRR integrated with a 2DGC. The 2DGC couples two orthogonal polarization states from a single mode optical fiber to the TE_0_ mode of two separate input waveguides of the 4-port MRR. The directional couplers are designed to overcouple light into MRR. The devices use two thermally tunable phase shifters: one phase shifter tunes the MRR resonance while the other tunes the phase difference between the two input bus waveguides. The tuning of the MRR can effectively control the output split ratio. The independent control of split ratio and interference between the input two bus waveguide enable the polarization state to be effectively manipulated as desired. The device was fabricated at the commercial foundry processes, and we used it to realize a multi-functional polarization processor, i.e., polarization descrambler, polarization switching, polarizer, and polarization analyzer (DOS and DOT). The experimental results for the polarization mode unscrambling achieved crosstalk levels below −21 dB for a 40 GHz high-speed optical communications signal. Polarization analyzer shows good performance configured either as a DOS or DOT analyzer.

## 2 Principle and device design

**Figure 1:**
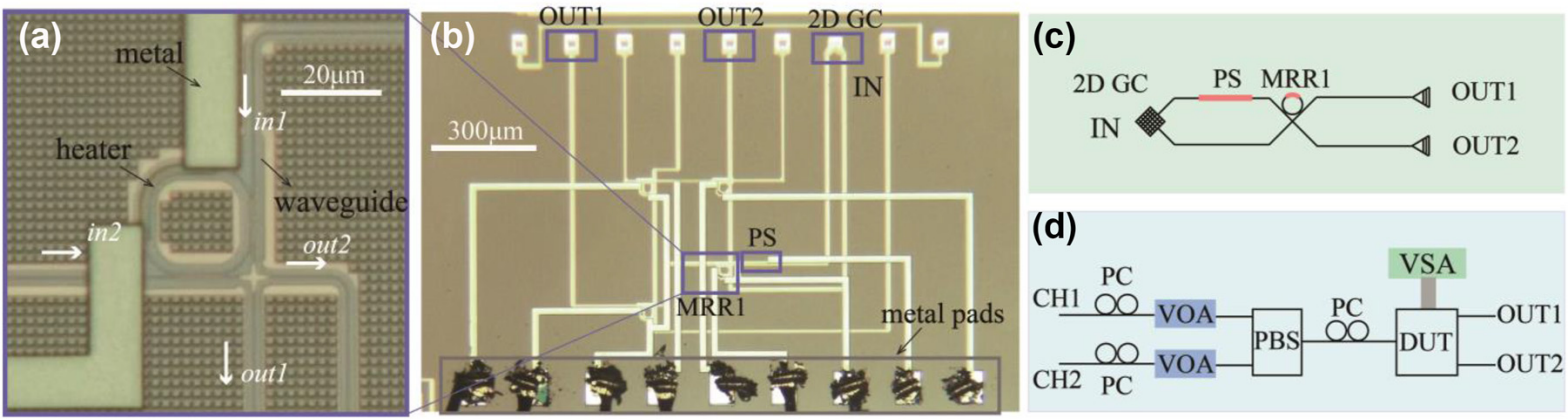
Micrograph image and schematics. (a) Micrograph image of the fabricated MRR. (b) Micrograph image of the proposed network. PS, GC represents phase shifter and grating coupler. (c) Schematic of the proposed network. (d) Experimental setup of the proposed system. PC, VOA, PBS, DUT, and VSA represent polarization controller, variable optical attenuator, polarization beam splitter, the device under test, and voltage source array.

The basic element of the proposed polarization polarizer is the 4-port coherent MRR as shown in Figure 1(a) and (b) and schematically in Figure 1(c). It comprises a dual-bus crossbar MRR. The ring waveguide is integrated with a thermally tunable phase shifter (PS) formed by a resistive metal heater embedded in the top oxide cladding above the ring waveguide. This PS is used to tune the resonance of the MRR. The MRR, therefore, can be regarded as a 2 by 2 lattice element providing a variable beam splitting ratio. The power coupling ratio from the bus waveguide into the MRR was designed to be between 0.3 and 0.4, and the radius of the ring waveguide is 10 μm. There are negligible bend losses in the single mode waveguide with this radius bend. We used a 2DGC to couple two orthogonal polarizations from the input single mode fiber. The 2DGC serves as a polarization diversity input as shown in Figure 1(b) and (c). The 2DGC couples light to the TE_0_ mode of the two input bus waveguides of the MRR. Another thermally tunable phase shifter was integrated to tune the phase difference between the two input waveguides. The 4-port MRR can be used to realize arbitrary 2 by 2 unitary matrix operations. The output optical field in the two output ports is produced by the interference between the two input waveguides and is also dependent on the splitting ratio as determined by the detuning of the ring resonator from its resonance. The 4-port MRR was fabricated by a commercial foundry (CUMEC) in a multi-project wafer (MPW) shuttle run. The MPW used 220 nm thick top silicon in a silicon-on-insulator (SOI) wafer with a buried oxide (BOX) of 2 μm. Micrograph images for the fabricated 4 port MRR, its input and output ports, and bond pads for the thermal phase shifters are shown in Figure 1(a) and (b), respectively. The width of the fully etched strip waveguide was designed to be 450 nm. The gap and coupling length of the directional coupler was designed to be 230 nm and 10 μm. The metal heater embedded in the oxide above the ring waveguide was designed to be 2.5 μm wide and 40 μm in length and had a measured resistance of 206 Ω. The heater length for the input waveguide PS was designed to be 2.5 μm wide and 80 μm in length and had a measured resistance of 408 Ω.

## 3 Experimental design

### 3.1 Device characteristics

**Figure 2:**
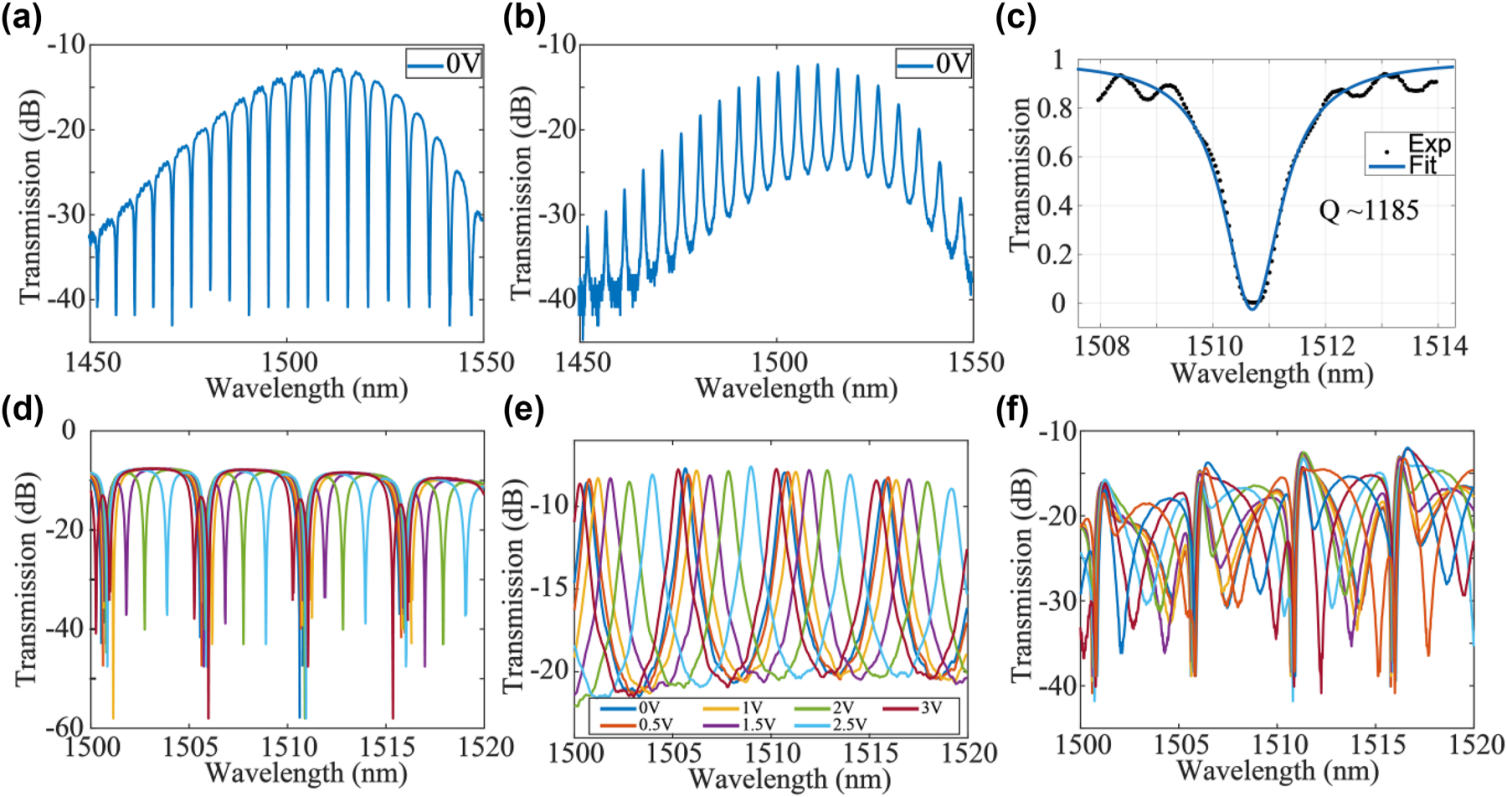
Device characteristics. (a) Transmission spectra of the through port of MRR. (b) Transmission spectra of the drop port of MRR. (c) Lorentz fit of the resonance of MRR, the quality factor was extracted to be 1185. (d) Transmission spectra of the through port of MRR and (e) transmission spectra of the drop port of MRR when applied with voltage from 0 V to 3 V. (f) Transmission spectra from port “IN” to port “OUT1” in Figure 1(c) when the PS was applied with a voltage range from 0 V to 3 V.

The experimental setup is shown in Figure 1(d). Light from a continuous-wave laser was split into two paths and used as the optical source for two transmission lanes formed by two polarization channels (CH1 and CH2). On each path, a polarization controller (PC) was used to adjust the polarization before combining the two channels using a fiber-optic packaged polarization beam splitter (PBS). In each path, we used a variable optical attenuator (VOA) to vary the power and measure the scattering matrix of the system. In this way, two independent data channels were coupled to the orthogonal modes in the single mode fiber. After the PBS, another PC enabled the setting of different test input polarizations orientations before coupling into the device under test (DUT). A simplified schematic of DUT is shown in Figure 1(c). Before applying the control algorithm on the two PS, the performance of the MRR and PS was characterized independently. Figure 2(a) and (b) shows the transmission spectra of the fabricated MRR, the resonance of the MRR was fitted with the Lorentz curve, and the loaded quality (*Q*) factor of the MRR was measured to be 1185. The tunability of the MRR is shown in Figure 2(d) and (e). The power consumption to tune the MRR over one free-spectral range was 59.5 mW. Figure 2(f) shows transmission spectra from port “IN” to port “OUT1.” With electrical power of 49.6 mW, the transmission spectra repeated, i.e., the input waveguide PS was tuned from 0 to 2π with 49.6 mW power.

### 3.2 Polarization descrambler and switching

**Figure 3:**
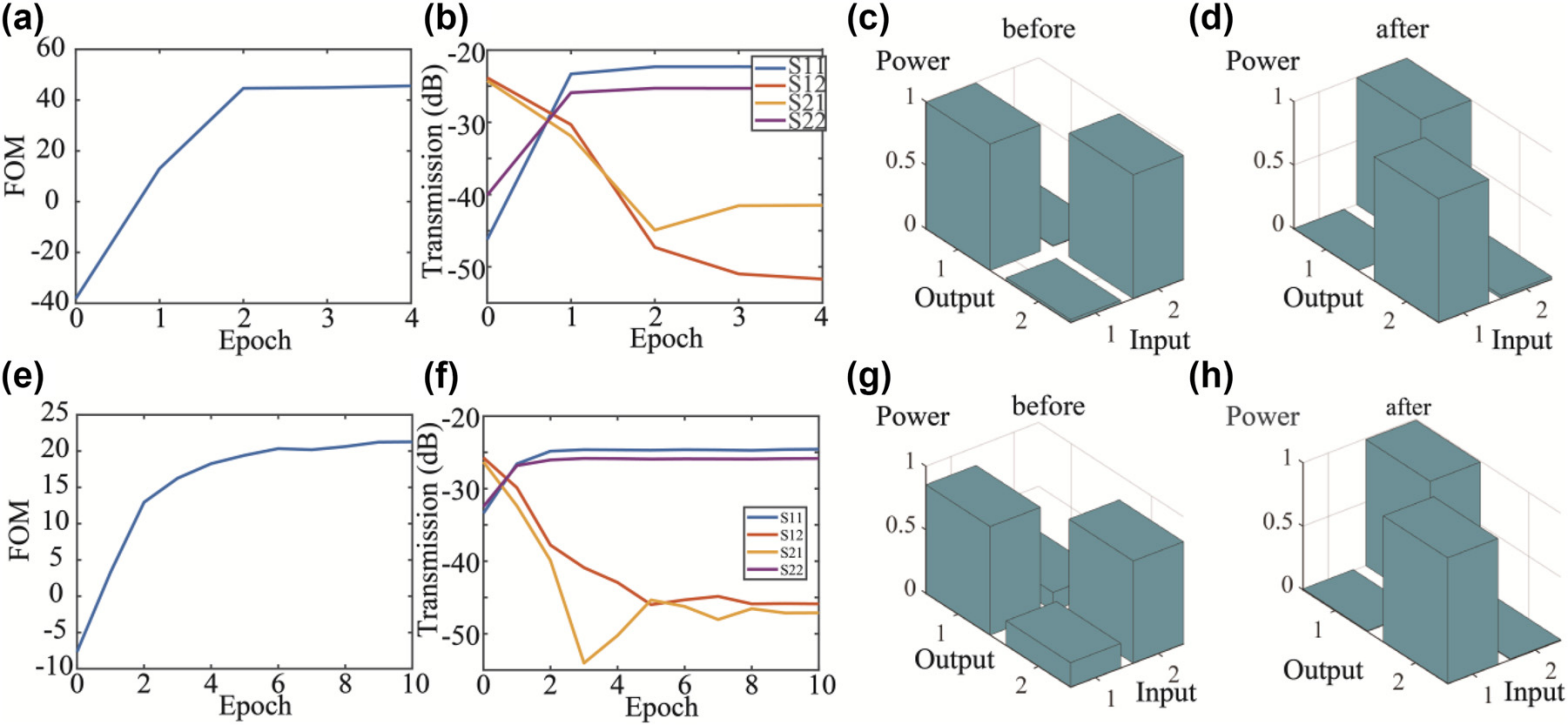
The training process of mode switching. (a) FOM value evolved with epoch number. (b) Scattering matrix evolved with epoch number. (c) Scattering matrix before switching and (d) scattering matrix after switching. (e–h) The training process of polarization unscrambling. (e) FOM value and (f) scattering matrix evolved with epoch number. Scattering matrix (g) before and (h) after polarization unscrambling.

We performed two experiments to demonstrate the polarization switching and the polarization mode unscrambling using the whole system, respectively. To facilitate the testing of polarization switching, the input fiber polarization controller was manually adjusted to maximize the coupling from TE mode into port “OUT1” (*S*_11_) and from TM mode into port “OUT2” (*S*_22_) as shown in Figure 3(c). To achieve polarization switching, we used particle swarm optimization to optimize the applied voltages on the PS and MRR in order to completely switch the output to the other output port “OUT2.” During the optimization process, we set the FOM = *S*_12_ + *S*_21_ − *S*_11_ − *S*_22_. The value of FOM evolved with optimization epochs are shown in Figure 3(a). *S*_*ij*_ represents power transmission from port “CH*i*” to port “OUT*j*”. Scattering matrix *S*_*ij*_ evolved with epochs was shown in Figure 3(b). FOM value was optimized from −40 dB to over 40 dB. This shows that the system can be configured as a polarization switch with polarization extinction ratio of 16 dB. The optimized scattering matrix is plotted in Figure 3(d) with crosstalk level < −16 dB. To achieve mode unscrambling, we used particle swarm optimization to optimize the applied voltages on the PSs. During the optimization process, we set the FOM = min((*S*_12_ − *S*_11_), (*S*_21_ − *S*_22_)). The value of FOM evolved with optimization epochs was shown in Figure 3(e). Scattering matrix *S*_*ij*_ evolved with epochs was shown in Figure 3(f). FOM value was optimized from −7 dB to 21 dB. The scattering matrix after optimization is plotted in Figure 3(d) with crosstalk level < −21 dB. This shows that two arbitrary input orthogonal modes in the fiber, irrespective of external disturbances and rotation in the fiber transmission, can always be unscrambled and restored to two different spatial outputs with crosstalk level lower than −21 dB in the proposed system. The same algorithm based on pilot tones can be used to adapt dynamically to changes in the state of polarization during the period of high-speed data transmission of a data packet. Continuous tracking of changes in the polarization state is limited by the maximum phase shift of about 2π in the integrated thermo-optic phase shifters, as there is a need to reset to zero phase shift beyond 2π, but the reset can be carried out during the initial setup for data packet transmission. The polarization processor can be suitable for data packet transmission such as the 100 G Ethernet standards used in data centers. The connection setup to establish the two lanes of data transmission can be established during the data link initiation protocol using the algorithms we mentioned above. The results also show that arbitrary polarization can be coupled to an optical fiber from the 2D GC if we operate the device in reverse and send light from port “OUT1” or port “OUT2” back to the 2DGC, because we can always tune the ratio and phase difference of the *x*- and *y*-polarization launched into the fiber.

### 3.3 High speed data transmission

**Figure 4:**
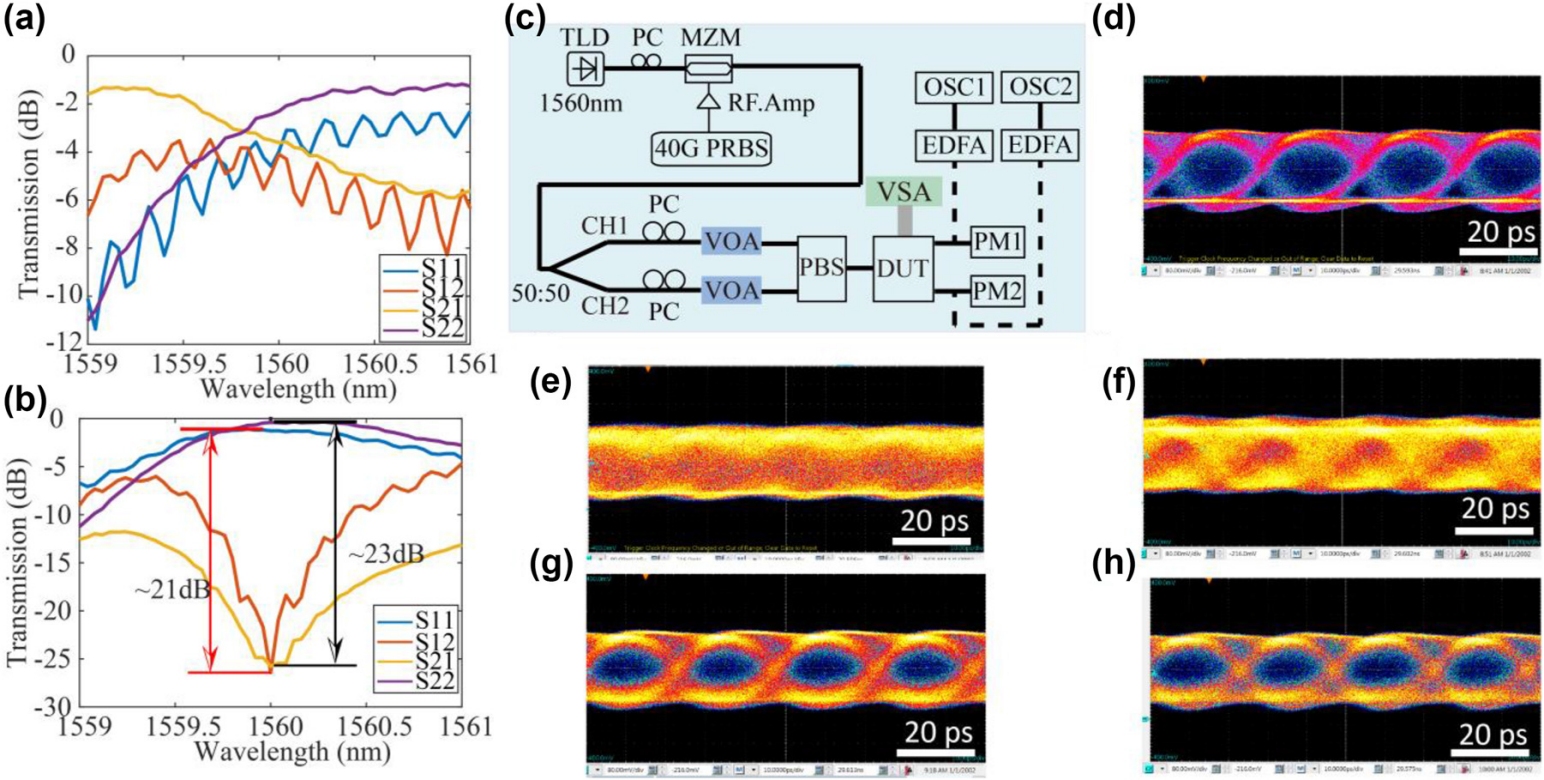
Transmission spectra of *S*_*ij*_ (a) before and (b) after being unscrambled by the coherent network. (c) Experimental setup of high-speed optical communication system using coherent network to do polarization unscrambling. TLD, MZM, PRBS, PM, EDFA, and OSC represent tunable laser diode, Mach–Zehnder modulator, pseudo-random binary sequence generation pattern, power monitor, erbium-doped fiber application amplifier, and high-speed oscilloscope. (d) Eye diagram generated by the MZM without going through the PIC system. Eye diagram before being scrambled by the optical system acquired by (e) OSC1 and (f) OSC2. Eye diagram unscrambled by the optical system acquired by (g) OSC1 and (h) OSC2.

One question we wish to address experimentally is to assess the limitation on signal bandwidth introduced by the use of the MRR in the polarization processor, and whether the polarization processor can be used to unscramble the pol-mux data lanes in a high-speed optical communication system. The important parameter in this application scenario is the optical bandwidth of the polarization processor. The optical bandwidth is determined by the optical path length difference within the system and the *Q* of the MRR (which had a loaded *Q* of 1185). Intersymbol crosstalk will be introduced if the delay time is too large. The system should have large bandwidth (small path length difference) if it was aimed at supporting high-speed data communication. The bandwidth of MRR Δ*f* can be estimated by the photon life time *τ* within the MRR by Δ*f* = 1/*τ*. Photon lifetime *τ* of an MRR can be derived from the *Q* of the MRR by *τ* = *n*_eff_*Qλ*/*πc*, where *n*_eff_ represents the effective index of waveguides, *λ* is the resonance wavelength. In this scenario, the system’s bandwidth was estimated to be around 200 GHz. To further investigate the bandwidth and the ability of the system to support high-speed communications, we carried out the experiment as shown in Figure 4(c). In Figure 4(c), we generated 40 GHz none-return-to-zero pseudo-random binary sequence signals and fed the two channels of pol-mux 40 GHz data into the optical fiber transmission link. On the receiver side, the two output channels were monitored by the power meters and the high-speed real-time oscilloscopes. Figure 4(a) and (b) shows the spectra of *S*_*ij*_ before and after being unscrambled by the MRR-based coherent network. Figure 4(d) shows the eye diagram generated by the Mach–Zehnder Modulator (MZM). Figure 4(e) and (f) shows the eye diagram acquired before being processed by the network, while Figure 4(g)–(h) shows the eye diagrams after the unscrambling of the data lanes by the integrated polarization processor. The unprocessed eye diagram is completely closed because the arbitrary choice of orthogonal polarization basis at the receiver does not match the original transmitter polarization basis and severe crosstalk is present in the two unprocessed output channels. However, after processing by the integrated 4-port MRR coherent network, the eye diagrams become opened by the reduction of crosstalk. Besides, we can initially observe that the shape of the eye diagram was not changed after transmitting through the optical system, which shows that the 4-port MRR processor can support high-speed optical communication at speed of at least 40 GHz. Furthermore, we applied this setup to showcase an all-optical transceiver system to implement PAM4 and polarization multiplexing in a direct-detection transceiver operating at 400 Gb/s [45]. The experimental results are consistent with system being transparent to both data rates and modulation formats for signals that occupy a frequency band under 200 GHz.

### 3.4 Polarization analyzer

#### 3.4.1 Division-of-space polarization analyzer

**Figure 5:**
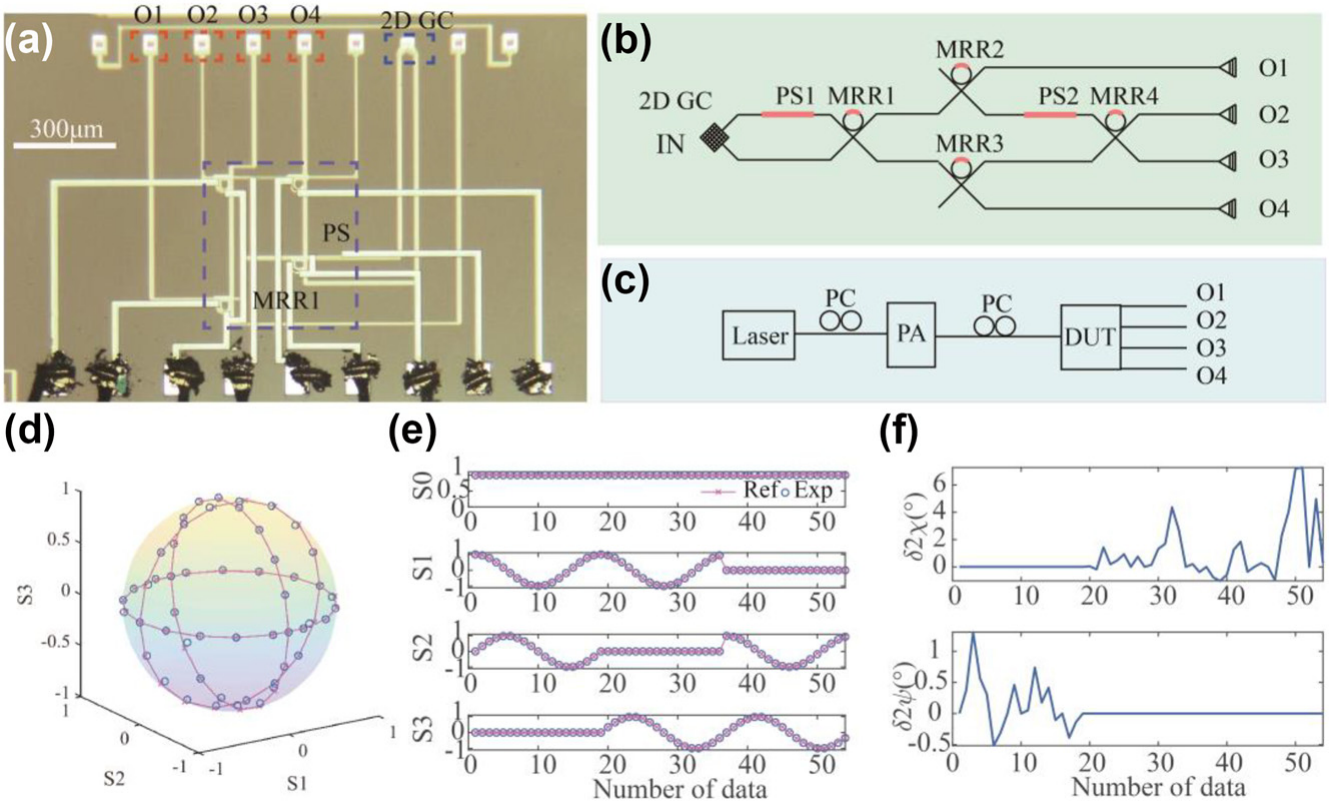
Division-of-space polarization analyzer. (a) Micrograph image and (b) schematic of the system when it was configured as a DOS polarization analyzer. (c) Experimental setup of the polarization analyzer. (d) The Poincare sphere shows the recovered polarization state. (e) The recovered Stokes parameters and (f) deviations of polarization parameters.

In this section, we show that the 4-port coherent MRR system can be configured as a DOS polarization analyzer. The schematic of the system is shown in Figure 5(a) and (b) while the experimental setup is shown in Figure 5(c). It can be proved that there exists a linear relationship between the Stokes parameters vector *S* and the four measured spatial outputs *I* (from O1 to O4) related by the matrix equation *S* = *TI*. The transmission matrix *T* can always be calibrated by four known polarization states. Then, any polarization state can be retrieved by measuring the spatial intensity vector *I*. The normalized Stokes parameter can be retrieved by *S* = *TI* and $\vec{S}=\left(S_{1},S_{2},S_{3}\right)/S_{0}$ . Figure 5(d) illustrates the Poincare sphere containing the recovered and reference points. The reference parameters are those defined by the commercial polarization generator/analyzer. Figure 5(e) depicts the recovered Stokes parameters, and Figure 5(f) shows the corresponding deviations from the reference parameters. One can see that the systems work well with only small measurement errors: the deviation of the orientation angle (2*ψ*) measured by the polarization analyzer varied from −0.5° to 1.3° and the deviation of ellipticity angle (2*χ*) varied from −0.98° to 7.27°.

#### 3.4.2 Division-of-time polarization analyzer

**Figure 6:**
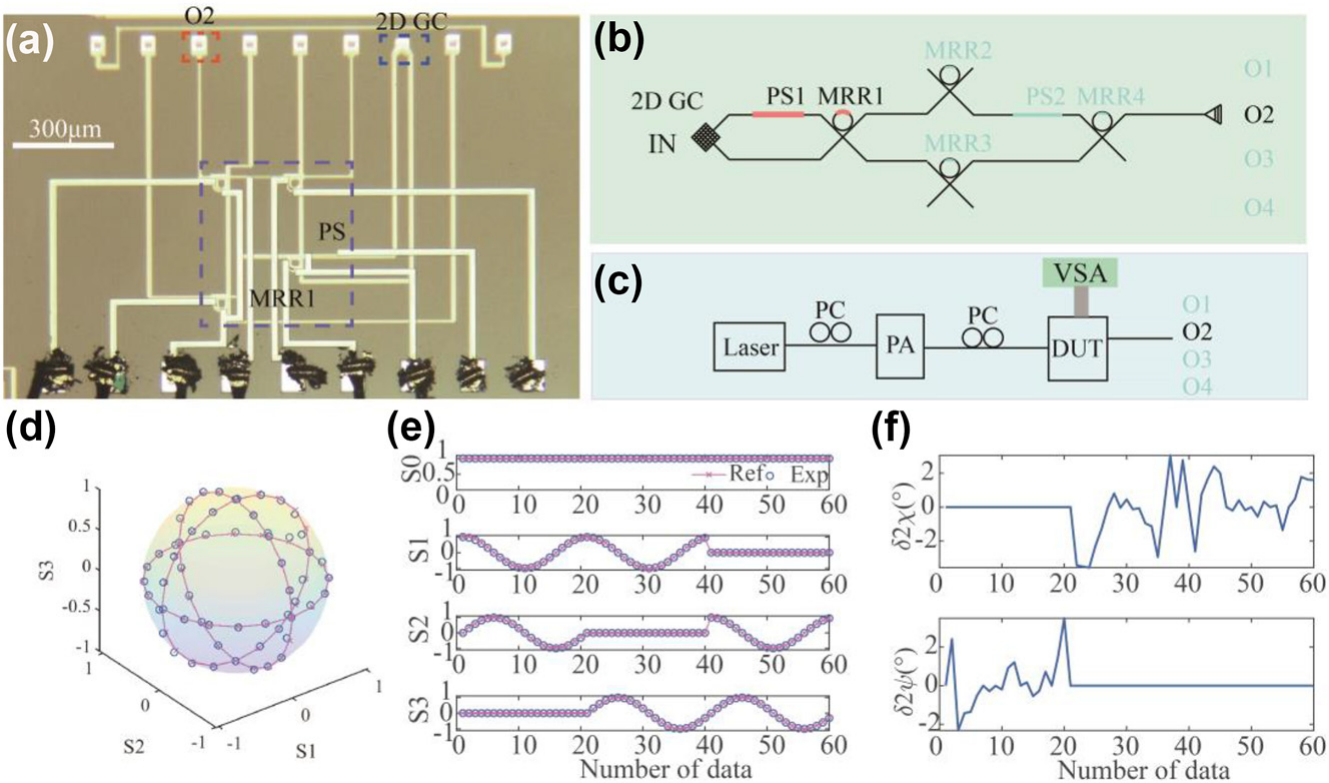
Division-of-time polarization analyzer. (a) Micrograph image and (b) schematic of the system when it was configured as a DOT polarization analyzer. (c) Experimental setup of the polarization analyzer. (d) The Poincare sphere shows the recovered polarization state. (e) The recovered Stokes parameters and (f) deviations of polarization parameters.

In this section, we show that the 4-port MRR system can be configured as a DOT PA. The schematic of the system is shown in Figure 6(a) and (b) while the experimental setup is shown in Figure 6(c). There exists a linear relationship between the Stokes parameters vector *S* and the four measured temporal output *I* (from O1) related by *S* = *TI*. The transmission matrix *T* can also be calibrated by four known polarization states. Then, any polarization state can be retrieved by measuring the temporal intensity vector *I*. The normalized Stokes parameter can be retrieved by *S* = *TI*, and Figure 6(d) illustrates the Poincare sphere containing the recovered and reference points. The reference parameters are those defined by a commercial polarization generator/analyzer. Figure 6(e) depicts the recovered Stokes parameters, and Figure 6(f) shows the corresponding deviations from the reference parameters. One can see that the measurement error of orientation angle relative to the reference is slightly worse than the DOS measurement: the DOT measurements had deviation of the orientation angle (2*ψ*) varied from −2.93° to 3.49°. However, the DOT measurement had slightly better performance than the DOS measurement for the ellipticity angle (2*χ*): the DOT measurement had deviation of ellipticity angle (2*χ*) that varied from −3.5° to 3.05°.

## 4 Discussion and conclusions

### 4.1 Discussion

Table 1 summarized and compared the state-of-the-art techniques that were utilized for polarization processing. Comparing with the traditional method using coherent receiver with DSP, the advantages of using integrated processor for polarization processor is lower latency and lower power consumption for the high data rate signals. Compared with the MZI-based integrated photonic processor, the MRR-based photonic processor has smaller footprint with rings of radius as small as 3 μm and possible of high-speed configuration without further sacrificing footprint. The other advantage is due to the smaller finesse (*F*) of MRR, the thermal power required to tune from 0 to 1 in MRR is finesse (*F*) times lower than that required for MZI. *F* is defined as the ratio of the FSR (Free Spectral Range) and the resonance full width at half maximum (FWHM), i.e., *F* = FSR/FWHM. It should be noted that normally *F* should be larger for MRRs compared to MZI and increases with the resonance *Q*-factor. The disadvantage of MRR is that the resonances are sensitive to fabrication errors. This is a well-known and well-studied problem. To address fabrication errors, there are three primary solutions commonly employed. One approach involves phase trimming [46] by laser annealing of germanium-implanted waveguides. Additionally, employing lithography with a higher resolution (<45 nm) can mitigate fabrication errors and enhance the consistency of MRR characteristics [47]. While these methods can reduce the spread of resonances of MRRs, thermal heaters are needed anyway to realize reconfigurable circuits. Notably, efforts have been undertaken to employ feedback loops for adaptive tuning of MRRs, aimed at mitigating the impact of environmental fluctuations [48].

**Table 1:** Comparison of different schemes for polarization processing.

Scheme	Footprint	Crosstalk	Speed	Bandwidth	Power consumption
Coherent receiver [49]	–	–	–	Limited by DSP	420 mW/Gbit/s
MZI [27]	∼100 μm in length	<−20 dB	KHz	Unlimited (within DC constraints)	*P*_FSR_
MRR [this work]	∼20 μm in radius	<−21 dB	KHz	<200 GHz	*P*_FSR_/*F*

Denote: *F* represents the finesse of MRR. DC represents the 3 dB splitters (directional couplers) that constrain the wavelength range of operation of MZI. *P*_FSR_ denotes the power required to tune across one free spectral range.

### 4.2 Conclusions

In conclusion, we have proposed and experimentally validated a novel high-performance polarization processor by using coherent 4-port MRR integrated with a 2DGC. The 4-port MRR can be used to tune the splitting ratio and phase difference between two polarization components and, therefore, can be configured to control and monitor the polarization states. The polarization processor can be used as polarization descrambler, polarization switch, polarizers, and polarization analyzer. This paper reports the first experimental demonstration of the coherent 4-port MRR for polarization mode switching and polarization mode unscrambling. We demonstrated how two pol-mux data channels can be recovered after transmission in nonpolarization maintaining optical fiber (introducing arbitrary polarization rotation during transmission) and that the integrated receiver can reduce the crosstalk level between the two data lanes to below −21 dB. These results show that pol-mux communications can be used for direct-detection optical fiber interconnects in data centers, without the need for using polarization maintaining optical fibers nor high-speed DSP to recover the transmitted polarization channels. The device demonstrates the successful use of the 4-port coherent network for matrix multiplications to process 40 GHz polarization-multiplexed communications signals. For the polarization analyzer, the DOS polarization analyzer achieved good accuracy in the measurement of the orientation angle (2*ψ*), with measurement errors varying from −0.5° to 1.3°, and reasonable accuracy in measurement of the ellipticity angle (2*χ*), with errors varying from −0.98° to 7.27°. The DOT polarization analyzer achieved the deviation of the orientation angle (2*ψ*) is from −2.93° to 3.49° and deviation of ellipticity angle (2*χ*) is from −3.5° to 3.05°.

## References

[1] Dai D., Bauters J., Bowers J. E. (2012). Passive technologies for future large-scale photonic integrated circuits on silicon: polarization handling, light non-reciprocity and loss reduction. *Light: Sci. Appl.*.

[2] Sun C., Wade M. T., Lee Y. (2015). Single-chip microprocessor that communicates directly using light. *Nature*.

[3] Hochberg M., Baehr-Jones T. (2010). Towards fabless silicon photonics. *Nat. Photonics*.

[4] Pfau T., Peveling R., Hauden J. (2007). Coherent digital polarization diversity receiver for real-time polarization-multiplexed QPSK transmission at 2.8 Gb/s. *IEEE Photon. Technol. Lett.*.

[5] Sjodin M., Johannisson P., Wymeersch H., Andrekson P. A., Karlsson M. (2011). Comparison of polarization-switched QPSK and polarization-multiplexed QPSK at 30 Gbit/s. *Opt. Express*.

[6] Dong P., Xie C., Chen L., Buhl L. L., Chen Y. K. (2012). 112-Gb/s monolithic PDM-QPSK modulator in silicon. *Opt. Express*.

[7] Li J., Zeng T., Li X. (2019). Real-time fast polarization tracking based on polarization phase locking least mean square algorithm. *Opt. Express*.

[8] Gundogan M., Ledingham P. M., Almasi A., Cristiani M., de Riedmatten H. (2012). Quantum storage of a photonic polarization qubit in a solid. *Phys. Rev. Lett*..

[9] Zahidy M., Liu Y., Cozzolino D. (2022). Photonic integrated chip enabling orbital angular momentum multiplexing for quantum communication. *Nanophotonics*.

[10] Corrielli G., Crespi A., Geremia R. (2014). Rotated waveplates in integrated waveguide optics. *Nat. Commun*..

[11] Ma C., Sacher W. D., Tang Z. (2016). Silicon photonic transmitter for polarization-encoded quantum key distribution. *Optica*.

[12] Kim J., Kwon J., Kim M., Do J., Lee D., Han H. (2016). Low-dielectric-constant polyimide aerogel composite films with low water uptake. *Polym. J.*.

[13] Howe J. D., Miller M. A., Blumer R. V. (2000). Polarization sensing for target acquisition and mine detection. *Polarization Analysis, Measurement, and Remote Sensing III*.

[14] Mecozzi A., Cantono M., Castellanos J. C., Kamalov V., Muller R., Zhan Z. (2021). Polarization sensing using submarine optical cables. *Optica*.

[15] Zhan Z., Cantono M., Kamalov V. (2021). Optical polarization-based seismic and water wave sensing on transoceanic cables. *Science*.

[16] Fukuda H., Yamada K., Tsuchizawa T., Watanabe T., Shinojima H., Itabashi S. i. (2008). Silicon photonic circuit with polarization diversity. *Opt. Express*.

[17] Xu H., Dai D., Shi Y. (2019). Anisotropic metamaterial-assisted all-silicon polarizer with 415-nm bandwidth. *Photon. Res.*.

[18] Wang B., Blaize S., Salas-Montiel R. (2019). Nanoscale plasmonic TM-pass polarizer integrated on silicon photonics. *Nanoscale*.

[19] Wang J., Zhao Y., Agha I., Sarangan A. M. (2015). SU-8 nanoimprint fabrication of wire-grid polarizers using deep-UV interference lithography. *Opt. Lett*..

[20] Taillaert D., Chong H., Borel P., Frandsen L., De La Rue R., Baets R. (2003). A compact two-dimensional grating coupler used as a polarization splitter. *IEEE Photon. Technol. Lett.*.

[21] Zhang Y., He Y., Wu J. (2016). High-extinction-ratio silicon polarization beam splitter with tolerance to waveguide width and coupling length variations. *Opt. Express*.

[22] Xu H., Dai D., Shi Y. (2019). Ultra-Broadband and ultra-compact on-chip silicon polarization beam splitter by using hetero-anisotropic metamaterials. *Laser Photon. Rev.*.

[23] Wu H., Tan Y., Dai D. (2017). Ultra-broadband high-performance polarizing beam splitter on silicon. *Opt. Express*.

[24] Dai D., Bowers J. E. (2011). Novel concept for ultracompact polarization splitter-rotator based on silicon nanowires. *Opt. Express*.

[25] Liu L., Ding Y., Yvind K., Hvam J. M. (2011). Silicon-on-insulator polarization splitting and rotating device for polarization diversity circuits. *Opt. Express*.

[26] Fukuda H., Wada K. (2014). Parallel-core-type polarization rotator for silicon wire waveguide platform. *Photon. Res.*.

[27] Zhou H., Zhao Y., Wei Y., Li F., Dong J., Zhang X. (2019). All-in-one silicon photonic polarization processor. *Nanophotonics*.

[28] Sarmiento-Merenguel J. D., Halir R., Le Roux X. (2015). Demonstration of integrated polarization control with a 40 dB range in extinction ratio. *Optica*.

[29] Zhao W., Liu R., Peng Y., Yi X., Chen H., Dai D. (2022). High-performance silicon polarization switch based on a Mach–Zehnder interferometer integrated with polarization-dependent mode converters. *Nanophotonics*.

[30] Sacher W. D., Barwicz T., Taylor B. J. F., Poon J. K. S. (2014). Polarization rotator-splitters in standard active silicon photonics platforms. *Opt. Express*.

[31] Guo D., Hou K., Tang W., Chu T. (2019). Silicon polarization switch based on symmetric polarization splitter-rotators. *J. Semicond.*.

[32] Wang Y., Li X., Jiang Z. (2021). Ultrahigh-speed graphene-based optical coherent receiver. *Nat. Commun*..

[33] Krummrich P. M., Ronnenberg D., Schairer W., Wienold D., Jenau F., Herrmann M. (2016). Demanding response time requirements on coherent receivers due to fast polarization rotations caused by lightning events. *Opt. Express*.

[34] Charlton D., Clarke S., Doucet D. (2017). Field measurements of SOP transients in OPGW, with time and location correlation to lightning strikes. *Opt. Express*.

[35] Waddy D. S., Chen L., Bao X. (2005). Polarization effects in aerial fibers. *Opt. Fiber Technol.*.

[36] Dong P., Chen X., Kim K., Chandrasekhar S., Chen Y. K., Sinsky J. H. (2016). 128-Gb/s 100-km transmission with direct detection using silicon photonic Stokes vector receiver and I/Q modulator. *Opt. Express*.

[37] Ghosh S., Kawabata Y., Tanemura T., Nakano Y. (2017). Polarization-analyzing circuit on InP for integrated Stokes vector receiver. *Opt. Express*.

[38] Wu W., Yu Y., Liu W., Zhang X. (2019). Fully integrated CMOS-compatible polarization analyzer. *Nanophotonics*.

[39] Lee K., Yun H., Mun S., Lee G., Sung J., Lee B. (2018). Ultracompact broadband plasmonic polarimeter. *Laser Photon. Rev.*.

[40] Yi D., Wu X., Tsang H. K. (2021). Ultra-compact polarization analyzer based on micro-ring resonators. *IEEE Photon. Technol. Lett.*.

[41] Shen Y., Harris N. C., Skirlo S. (2017). Deep learning with coherent nanophotonic circuits. *Nat. Photonics*.

[42] Harris N. C., Carolan J., Bunandar D. (2018). Linear programmable nanophotonic processors. *Optica*.

[43] Annoni A., Guglielmi E., Carminati M. (2017). Unscrambling light-automatically undoing strong mixing between modes. *Light Sci. Appl.*.

[44] Yi D., Wang Y., Tsang H. K. (2021). Multi-functional photonic processors using coherent network of micro-ring resonators. *APL Photonics*.

[45] David W. U. C., Yi D., Tong Y., Chow C.-W., Tsang H. K. (2023). Towards 3.2 Tb/s direct detection polarization-multiplexed transceivers using on-chip microring assisted coherent network. *ECOC*.

[46] Chen X., Milosevic M. M., Thomson D. J. (2017). Post-fabrication phase trimming of Mach–Zehnder interferometers by laser annealing of germanium implanted waveguides. *Photon. Res.*.

[47] Stojanović V., Ram R. J., Popović M. (2018). Monolithic silicon-photonic platforms in state-of-the-art CMOS SOI processes [Invited]. *Opt. Express*.

[48] Zanetto F., Grimaldi V., Moralis-Pegios M. (2020). WDM-based silicon photonic multi-socket interconnect architecture with automated wavelength and thermal drift compensation. *J. Lightwave Technol.*.

[49] Bernini A. W., Fice M. J., Balakier K. (2022). Low-power-consumption coherent receiver architecture for satellite optical links. ..

